# Influence of Acidic Storage and Simulated Toothbrushing on the Translucency and Color Stability of 3D-Printed Resins for Prosthodontic Applications

**DOI:** 10.3390/ma18173942

**Published:** 2025-08-22

**Authors:** Sarah M. Alnafaiy, Nawaf Labban, Alhanoof Saleh Aldegheishem, Saleh Alhijji, Refal Saad Albaijan, Saad Saleh AlResayes, Rafa Abdulrahman Alsultan, Abeer Mohammed Alrossais, Rahaf Farhan Alanazi

**Affiliations:** 1 Department of Clinical Dental Sciences, College of Dentistry, Princess Nourah bint Abdulrahman University, P.O. Box 84428, Riyadh 11671, Saudi Arabia; asaldegheishem@pnu.edu.sa; 2 Department of Prosthetic Dental Sciences, College of Dentistry, King Saud University, P.O. Box 60169, Riyadh 11545, Saudi Arabia; nalabban@ksu.edu.sa (N.L.); salresayes@ksu.edu.sa (S.S.A.); 3 Department of Dental Health, College of Applied Medical Sciences, King Saud University, P.O. Box 10219, Riyadh 12372, Saudi Arabia; smalhijji@ksu.edu.sa; 4 Department of Prosthodontics, College of Dentistry, Prince Sattam bin Abdulaziz University, P.O. Box 173, Al-Kharj 11942, Saudi Arabia; r.albaijan@psau.edu.sa; 5 College of Dentistry, Princess Nourah bint Abdulrahman University, P.O. Box 84428, Riyadh 11671, Saudi Arabia; alsultanrafa2@gmail.com (R.A.A.); abeer.rossais@gmail.com (A.M.A.); rahfaalanazi@gmail.com (R.F.A.)

**Keywords:** 3D printing, CAD/CAM, color, dental ceramics, tooth brushing, translucency

## Abstract

This study aimed to assess the effect of acidic storage and simulated brushing on the translucency and color stability of 3D-printed resins for prosthodontic applications. Three 3D printed resin materials—Ceramic Crown (CC), OnX (ONX), and Tough 2 (T2)—were compared with a CAD/CAM milled nano-ceramic resin material (Lava Ultimate, LU). Twelve specimens were fabricated from each material and were allocated into two groups based on the storage medium (water or citric acid), followed by simulated tooth brushing for 3650 cycles. The specimens’ translucency (TP) and color stability (ΔE) were determined using a spectrophotometer. The data was compared using ANOVA, independent student t-tests, and a post hoc Tukey test (*p* < 0.05). Multiple comparisons of mean differences in TP revealed significant differences between the tested materials (*p* < 0.001), except for groups CC and ONX. Irrespective of the groups, all materials showed decreased TP values after simulated tooth brushing. Regarding color stability, CC (0.66 ± 0.42) and T2 (1.40 ± 0.34) in acid demonstrated the least and greatest color changes, respectively. The ΔE did not vary between the materials or between the storage media (*p* > 0.05). Except for T2 and LU in water, the other materials showed ΔE values below the perceptibility threshold of 1.2. The material type and storage media affected the translucency of the tested materials. However, regardless of the material type and storage media, there was no discernible impact on the color change of the tested materials.

## 1. Introduction

Over the past thirty years, significant development in computer-aided design and computer-aided manufacturing (CAD/CAM) for dental restorations has led to the widespread adoption of this technology in routine clinical practice. This technology has transformed the fabrication process of dental restorations, enabling more precise and efficient outcomes [1]. The most common CAD/CAM digital technologies that have been used in dental practice are milling and 3D printing [2]. In CAD/CAM milling, dental restorations are milled from blocks, while dental 3D printing uses a light or laser to polymerize liquid materials with high accuracy to fabricate restorations [3].

The use of CAD/CAM milling is limited by material waste, the limited movement range of the cutting devices, wear of the milling burs, and the inability to recycle material waste [4]. On the contrary, the layer-by-layer additive process of 3D printing offers greater design flexibility, which allows for creating complex anatomical geometries and internal structures that are difficult to achieve with milling [5]. Additionally, 3D printing enables faster production by fabricating multiple restorations simultaneously, thereby reducing production time compared to milling [3,6]. The advent of 3D-printing technology has significantly expanded its use by introducing new materials for permanent or long-term dental restorations [7]. Despite the many advantages of 3D printing, the intrinsic properties of the resins pose problems compared to more conventional and CAD/CAM materials. Therefore, advancements in dental materials and procedures are necessary for maximizing the full potential of 3D printing [8,9].

The volume of the filler is one of the primary drawbacks of printable resins. It has been demonstrated that incorporating excess filler may hinder the flow of the resin during the fabrication process, increasing the likelihood of air bubbles or the formation of non-homogeneous microstructure areas, thereby compromising the mechanical qualities. Therefore, compared to resins made for subtractive manufacture (80–85 wt%), the printable resins that are now on the market include a substantially lower amount of filler (30–50 wt%) [9,10]. In recent years, researchers have resorted to nanoparticle reinforcement of the polymer matrix, which has remarkable resilience to corrosion and durability, making them extremely promising. Furthermore, nanoparticle characteristics can be further optimized by adjusting their size, along with the shape and nature, which have an enormous effect on the properties of the resulting material [8,9].

Binder modification is another method of enhancing the characteristics of dental materials [11]. Multiple studies have been conducted to substitute the hydroxyl groups with different side groups in order to develop a low-viscosity bisphenol A-glycidyl methacrylate (bisGMA) resin with minimal polymerization shrinkage [12]. Pereira et al. [13] substituted fluoromethyl and methyl groups for the core and side, respectively, whereas Kim et al. [14] substituted methoxy groups for hydroxyl groups. Another investigation by Srivastava et al. involved esterifying bisGMA using different chlorides of aliphatic acids [12]. A new polymeric system that is distinct from the conventional methacrylate systems has also been investigated as a potential substitute to develop resin materials that are significantly superior. The organic matrix of resin composites has been modified by a variety of polymer chemistries, including siloranes, liquid crystals, isobornyl acrylate, and thiolenes [15].

A relatively new hybrid material on the market, resin matrix ceramic (RMC), aims to combine the advantages of composite resins for polishability and intraoral reparability with the color stability and strength of ceramics. CAD/CAM technology via milling is primarily used for the fabrication of dental prosthesis using RMCs [16]. Polymer-infiltrated ceramic-network (PICN) and nano-ceramic resin (NCR) materials are the two types of RMC materials used for milled prostheses. PICN materials comprise a ceramic network infiltrated with organic polymer (Vita Enamic; VITA Zahnfabrik, Bad Säckingen, Germany), while NCR comprise a polymer matrix with inorganic nano-ceramic fillers (Lava Ultimate; 3M ESPE, St. Paul, MN, USA and Cerasmart; GC America Inc., Alsip, IL, USA) [16,17]. The incorporation of nano-sized or nano-fused ceramic particles into the polymer matrix is reported to enhance the mechanical properties of the materials [6,18]. On the other hand, manufacturers have suggested that particles in 3D-printed hybrid ceramics display a strengthening effect between the organic resin matrix and inorganic ceramic fillers, improving their mechanical and optical properties [19].

One of the common treatment options for people who are completely edentulous and undergoing rehabilitation is hybrid dentures. Multiple implants supporting these dentures provide stability, retention, and load distribution during chewing. Compared to conventional removable dentures, they provide several benefits, including better function, aesthetics, patient comfort, and long-term bone preservation. Since they seamlessly combine functional stability with realistic visual appeal, they are especially beneficial for patients with strong smile lines or those with high expectations for appearance [20,21].

In the evolving prosthodontic dental specialty, new materials for dental applications have been developed using 3D-printing technology. In recent years, several NCR hybrid materials for 3D printing have been introduced to the market since their breakthrough invention in early 2020 [22]. These materials are used for the fabrication of permanent crowns, inlays, onlays, veneers, and implant-supported hybrid prostheses [22]. In that context, OnX is a nano-ceramic hybrid 3D-printing resin, indicated for provisional implant-supported fixed hybrid denture and denture teeth, combining translucency, opacity to closely mimic natural dentition and the strength of ceramics, and high bond strength of the resin to the denture base [23]. Tough 2, an FDA-cleared 3D-printed resin developed using cutting-edge nanofusion technology, is used for the fabrication of implant-supported fixed hybrid dentures. This technology allows dental professionals to fabricate implant-supported fixed hybrid dentures chairside, combining high mechanical properties with a natural appearance [24]. Ceramic Crown is a chair-side nano-ceramic 3D-printing resin developed for full and partial crowns, veneers, and more, with more than 50% inorganic ceramic fillers, providing high radiopacity and exceptional esthetics that seamlessly integrate with natural teeth [25]. All these materials have been recently introduced to the market from a single manufacturer (https://sprintray.com/, accessed on 22 November 2024).

Previous studies on 3D-printed materials have largely focused on conventional and CAD/CAM denture base materials or crown and bridge materials. In the past few years, patients’ preference for hybrid denture materials has significantly increased. It is therefore essential to investigate the performance of new materials used for hybrid dentures, especially the changes in optical properties when exposed to environmental factors or with long-term use [26]. The optical characteristics, such as translucency, are critical in mimicking the natural appearance of teeth [27,28]. Translucency determines the behavior of light in an object through the phenomena of light transmission, scattering, and absorption, which aids in the color perception of dental materials [29]. Studies have reported that prolonged exposure to the acidic oral environment due to carbonated drinks, in addition to vigorous brushing, increases surface roughness and discoloration with ceramic resin materials, thereby impairing their esthetic appearance [30,31]. Although several studies have evaluated the optical properties of milled NCR materials, there is limited evidence of information regarding the optical properties of the newly introduced nano-ceramic reinforced or nano-fused 3D-printed resin [2,18,32].

Therefore, this study assessed the effect of acidic storage and simulated brushing on the translucency and color stability of three 3D-printed and one CAD/CAM-milled resin. It was hypothesized that there would be no significant difference in the translucency and color changes of the tested materials exposed to acidic storage and simulated tooth brushing.

## 2. Materials and Methods

### 2.1. Materials

Three 3D-printing NCR materials—Ceramic Crown, OnX, Tough 2, and a CAD/CAM milling material (Lava Ultimate; control)—were tested in this study. Table 1 presents the composition and manufacturers’ details of the study materials.

### 2.2. Specimen Preparation

**Figure 1:**
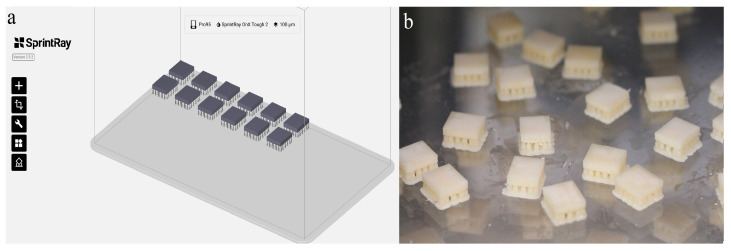
(**a**) Specimen designed using the CAD software; (**b**) 3D-printed specimen.

The sample size was estimated based on a previous similar study with an effect size of 0.35 [30], power of 0.80, and 5% alpha error (α-0.05), which required at least 12 specimens in each group. Twelve rectangular specimens were prepared from each material with a specified dimension of 12 × 14 × 2.5 mm ± 0.05. The specimens for LU were prepared by sectioning the CAD/CAM blocks to the desired thickness using an automated saw (IsoMet 1000 Precision; Buehler, Lake Bluff, IL, USA) under water coolant. For the 3D-printing resin materials, the specimen was designed using CAD software (RayWare 2.8.3, SprintRay Cloud Design; SprintRay Inc., Los Angeles, CA, USA) and saved as a Standard Tessellation Language (STL) file (Figure 1a). The STL file was exported to the print software, and the specimens were printed using a 3D printer (SprintRay Pro S; SprintRay Inc., Los Angeles, CA, USA) at 50 µm layer thickness and 0° printing orientations (Figure 1b). All specimens were then washed (ProWash S; SprintRay Inc., Los Angeles, CA, USA) and dried for 9 min. The specimens were then post-cured for 5 min (ProCure 2; SprintRay Inc., Los Angeles, CA, USA).

### 2.3. Specimen Polishing

All the specimens were ground with sequential use of 400-, 600-, 800-, and 1200-grit silicon carbide abrasives (LECO spectrum system, Bloomfield, CT, USA) under a water coolant. Next, a low-speed hand piece equipped with fine and medium-sized rubber wheels (red and green rubber wheels; Dedeco International, Inc., Long Eddy, NY, USA) at 10,000 rpm combined with a diamond polishing paste (Meta Di Supreme; Buehler Co., Lake Bluff, IL, USA) was used for final finishing of the specimens.

### 2.4. Specimen Allocation and Treatment

**Figure 2:**
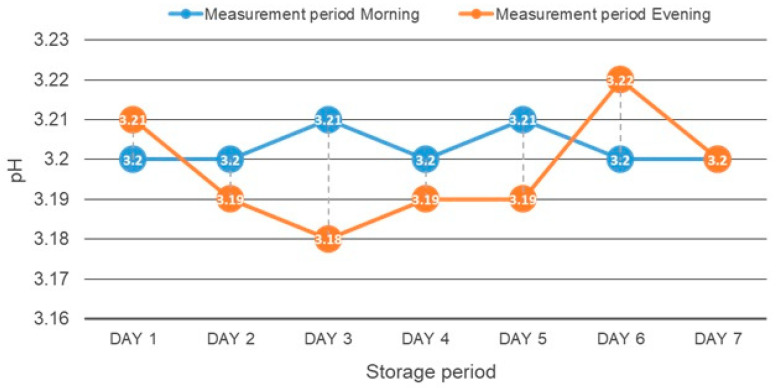
pH trend of the acidic storage during the immersion period.

The specimens from each material were divided into two groups based on the storage medium: distilled water (*n* = 6) and 0.3% citric acid (pH 3.2) (*n* = 6). All the specimens were stored for 7 days in their respective solutions in an incubator at 23 °C. The solutions were refreshed, and the pH of the solutions was continuously monitored twice daily (morning at 11 a.m. and evening at 7 p.m.) every day till the conclusion of the immersion period (Figure 2).

Subsequently, following the storage period, the specimens were removed from the solutions and thoroughly washed for 10 min. Next, the specimens were exposed to simulated brushing in a tooth-brushing machine (V-8; Omron, Kyoto, Japan). Each specimen was fixed onto the individual specimen container and filled with a slurry of toothpaste and distilled water, mixed at a 1:3 ratios. Toothbrushes (Oral-B P40; Procter and Gamble, Cincinnati, OH, USA) were attached to the individual slots, and each specimen was subjected to 3650 brushing cycles (7300 tooth brushing strokes) that equaled one year of intra-oral use [33].

### 2.5. Measurement of Color and Translucency

Spectrophotometric color analysis of the specimens was performed before and after the surface treatment. The L*, a*, and b* color values for each specimen were determined against a black and white background using a spectrophotometer (Labscan XE; hunter associates laboratory Inc., Reston, VA, USA). The spectrophotometer with an inbuilt integrated sphere with D65 illumination curve and 10° observation angle was operated at a wavelength of 360–740 nm [34]. Translucency was assessed using the translucency parameter (TP) by comparing the L*, a*, and b* measurements over white and black backgrounds using the equation [28,35]:

(1) TP=Lw*−Lb*2+aw*−ab*2+bw*−bb*212

where L is the difference between the light and dark color, a is the difference in red and green chromatic scale, and b is the difference in quantity of yellow and blue chromatic scale [36]. The subscripts w and b refer to color coordinates over white and black backgrounds, respectively.

According to the International Organization for Standardization (ISO) technical specification ISO/TR 28642:2016, the color difference (ΔE) of the specimen was quantified using the CIELab equation [37]:

(2) $$\Delta E=[({∆L}^{*}{)}^{2}+({∆a}^{*}{)}^{2}+({∆b}^{*}{)}^{2}{]}^{1/2}$$

**Figure 3:**
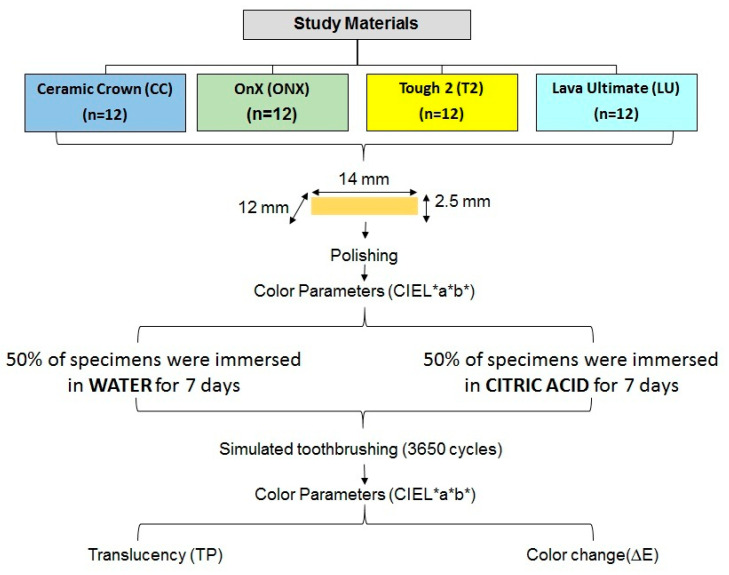
Study process.

ΔL*, Δa*, and Δb* are the differences between the pre- and post-surface treatment L*, a*, and b* values. The reported perceptibility threshold (PT) and acceptability threshold (AT) color values in dentistry are ∆E = 1.2 and ∆E = 2.7, respectively [38]. Any ∆E > 2.7 is clinically unacceptable. Figure 3 presents the study process chart of the present study.

### 2.6. Scanning Electron Microscopy (SEM) Evaluation of the Specimens

Specimens from both the water and acid groups were qualitatively assessed at baseline and after simulated tooth brushing using SEM (JSM-6610LV, JEOL Ltd., Tokyo, Japan). The specimens were gold sputter-coated, and the images were obtained at a working distance of 10 µm, 20 kV power, and ×1000 magnification.

### 2.7. Data Analysis

Data was analyzed using SPSS v.22 (IBM SPSS Inc., Chicago, IL, USA). Descriptive analysis included the expression of TP and ∆E values in terms of mean ± standard deviation (SD) for each group. For inferential statistics, a two-way ANOVA test was performed to observe the interaction between the materials and storage medium on TP and ∆E values. The mean TP and ∆E values between different materials for each storage medium were compared using one-way ANOVA. An independent Student *t* test was used to compare the mean TP and ∆E values between the storage medium for each material (*p* = 0.05).

## 3. Results

### 3.1. Translucency Parameter (TP)

Two-way ANOVA demonstrated that material type and the interaction between the material and storage media had a significant effect on TP (*p* < 0.001). However, the storage media independently had no significant effect on the TP (Table 2).

The mean TP of the tested materials at baseline and after simulated brushing is presented in Table 3. At baseline, the lowest translucency was demonstrated by material ONX (TP =2.55–2.64), followed by CC (TP = 2.79–3.20) and T2 (TP = 4.91–4.93). The highest translucency was observed with LU (TP = 7.23–7.49). After simulated brushing, the highest and lowest translucencies in the water group were found in CC (1.96 ± 0.12) and LU (7.51 ± 0.64), respectively. On the contrary, the highest and lowest TPs in the acid group were found in ONX (1.97 ± 0.22) and LU (7.04 ± 0.59), respectively. Irrespective of the groups, all materials showed a decrease in TP values from baseline to after simulated brushing.

The comparison between the materials at baseline and after simulated brushing showed a significant difference in TP values (*p* < 0.001). On the contrary, the comparison of TP within the materials from baseline to after simulated brushing for both groups was not statistically significant (*p* > 0.05). Multiple comparisons of mean differences in TP values between the materials for both groups at baseline and after simulated brushing revealed a significant difference between the materials (*p* < 0.001), except between CC and ONX materials in both groups (*p* > 0.05) (Table 4).

### 3.2. Color Stability (ΔE)

Two-way ANOVA demonstrated that the material type, the storage media, or the interaction between the material and the storage media did not significantly impact the ΔE (*p* > 0.05) (Table 5).

**Figure 4:**
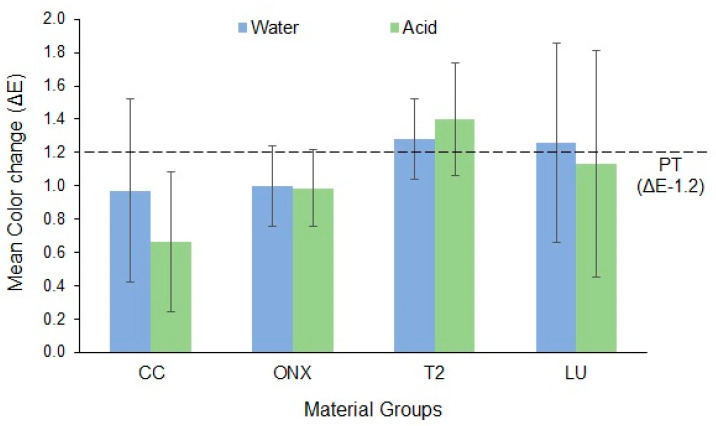
Mean color change (ΔE) of the materials, PT—perceptibility threshold.

The mean and SD for the ΔE of the tested materials for both groups are presented in Figure 4. The materials’ CC (0.66 ± 0.42) and T2 (1.40 ± 0.34) in the acid group demonstrated the least and highest color changes, respectively. All the materials, except T2 and LU in water, showed ΔE values below the perceptibility threshold of 1.2.

Table 6 presents the comparison of ΔE between the materials after simulated brushing. The results showed that the ΔE did not vary between the materials or between the storage media (*p* > 0.05).

**Figure 5:**
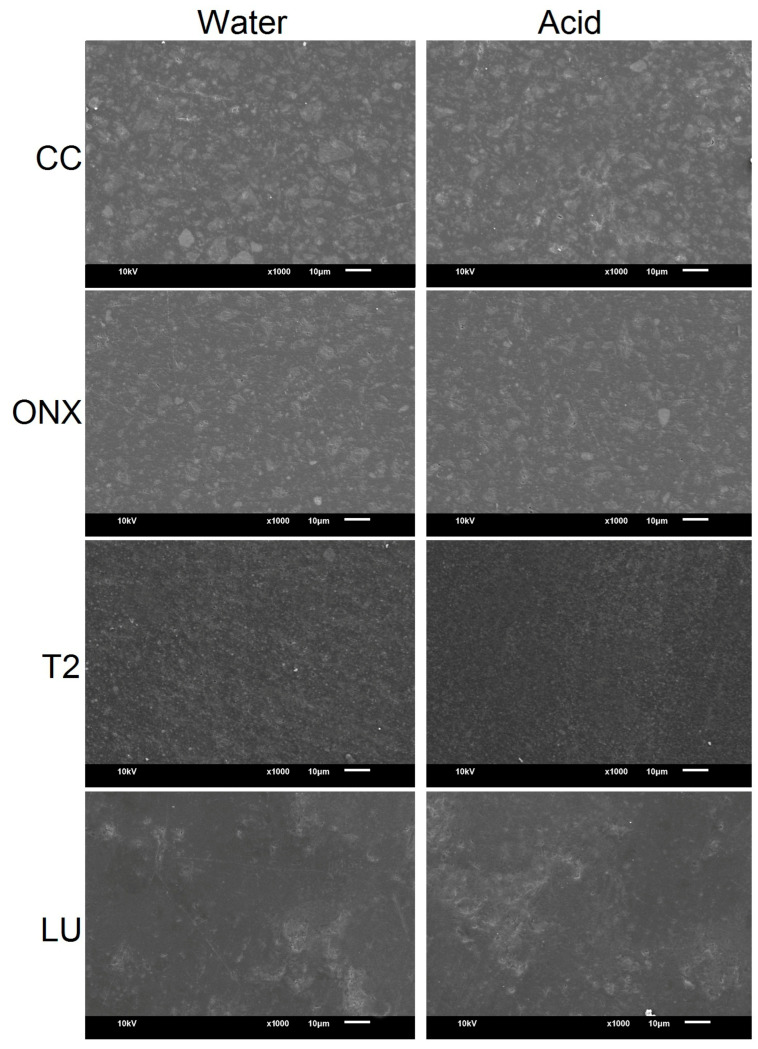
Scanning electron microscope image (×1000 magnification) of the materials at baseline.

**Figure 6:**
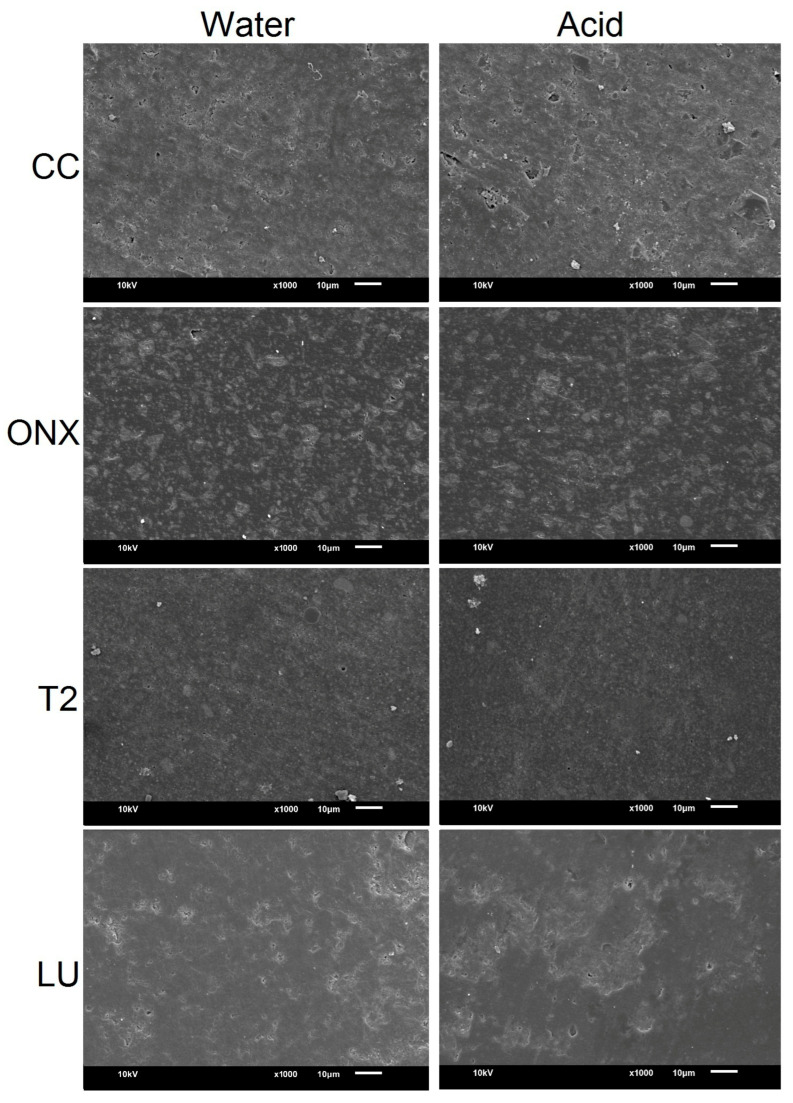
Scanning electron microscope image (×1000 magnification) of the materials after simulated brushing.

The SEM images at baseline and after simulated brushing are presented in Figure 5 and Figure 6, respectively. Baseline SEM images (Figure 5) showed substantial differences regarding the NCR materials, with CC and ONX demonstrating scattered filler particles. However, the T2 material comparatively presented a more homogeneous surface. The surface of LU was comparable with CC and ONX materials. After immersion in storage media and simulated brushing (Figure 6), CC material in acid demonstrated significant surface changes compared to the baseline and with other materials in the form of erosive craters and grooves. The ONX surface showed a more pronounced and coarser surface than the baseline specimens, but no substantial difference was observed between the water and acid groups. The surface of LU was almost comparable with the CC material, with the presence of rough, irregular granular surfaces. Among the materials, T2 was relatively even and smooth compared to other materials in both storage media.

## 4. Discussion

The current study assessed the effect of acidic storage and simulated brushing on the translucency and color stability of three 3D-printed and one CAD/CAM-milled hybrid nano-ceramic resins. The TP values revealed a significant difference between the tested materials, except between 3D-printed CC and ONX hybrid nano-ceramic resins. On the contrary, the acidic storage and simulated brushing did not affect the ΔE of the materials. Accordingly, the study’s hypotheses that stated there is no significant difference in the translucency and color changes of the tested materials exposed to acidic storage and simulated tooth brushing were partially rejected.

When selecting dental materials, the susceptibility of the esthetic dental restorative material to translucency and color change needs consideration. This is important for the appearance of and patient satisfaction with the restoration, especially in the anterior region [39]. Furthermore, the potential discoloration of the dental restorative material over time, due to exposure to water, an acidic environment, and mechanical brushing, is a crucial factor that requires evaluation [18]. Translucency refers to a material’s capacity to transmit and diffuse light. Translucency is crucial in restorative dentistry because it replicates the optical characteristics of natural teeth, which vary in translucency according to their thickness and location. Materials with a high degree of translucency are necessary for CAD/CAM technologies to produce realistic restorations [40]. Translucency can be assessed using different approaches, such as the TP, TP00, and contrast ratio (CR). A direct indicator of translucency, the translucency parameter (TP), is the color difference of a material at a given thickness against natural black and white backgrounds [31,41].

In the current study, the baseline TP values ranged between 2.55 and 7.49 (LU > T2 > CC > ONX), with CAD/CAM milled LU and 3D-printed ONX demonstrating the highest and lowest values. Following simulated brushing, specimens in the water group showed values between 1.96 and 7.51 (LU >T2 > CC > ONX), and the specimens in the acid group showed values between 1.97 and 7.04 (LU > T2 > ONX > CC). All the materials, irrespective of the groups, showed reduced TP values from baseline to simulated brushing except LU in water, which demonstrated increased TP. However, the increase in the TP of LU was negligible, non-significant, and considered clinically non-relevant. It has been reported that the texture of the material surface is altered by a combination of tooth abrasion and changes in the oral environment. A mismatched refractive index between the ceramic and resin fillers causes the porous and roughened surface to alter light refraction, which lowers translucency and the perceived color [30]. Translucency of the tested materials in this study differed between the 3D-printed and milled materials, and furthermore, the TPs varied between the 3D-printed materials.

A material’s color appearance is a complex synthesis of its optical qualities, especially its light transmittance. The impact of filler features, including size, shape, and content, on the light transmittance properties of restorative materials has been documented in earlier research. Additionally, it has been demonstrated that the optical characteristics of the restorative material are greatly impacted by the filler’s content and particle size [30,42]. The a* and b* values of materials with spherical and irregular fillers differed significantly. When fillers have irregular shapes, the a* value decreases and the b* value increases with an increase in filler quantity. On the contrary, with spherical-shaped filler, the a* value increases and the b* value decreases [42]. Furthermore, the smallest filler size has the highest overall light transmittance values for all filler contents, whereas larger fillers exhibit lower light transmittance values.

Among the tested materials, LU demonstrated increased TP. The silica/zirconia nanoparticles (80%) incorporated in a strongly cross-linked resin matrix may be a possible explanation for the increased TP values of the LU. Translucency is improved by nanometer-sized filler particles, which have diameters smaller than the visible-light wavelength. This results in less light scattering and enhanced light transmission [16]. Although the materials showed a significant difference in TP, the acid group was no different from the water group in terms of TP values. This infers that these materials were not affected by the acidic pH. It is expected that dental restorative materials have TP values near the enamel, which is reported to be 15–19 for a 1 mm specimen [43]. Gunal and Ulusoy [44] reported TP values of 17.93 for a 1 mm-thick specimen of LU. Vichi et al. [40] demonstrated a non-significant difference in the TP values between 1 mm thick LU (12.93 ± 0.32) and 3D-printed resin (permanent crown resin) (12.52 ± 0.19) specimens, which was in disagreement with the current study’s outcome. Kim et al. [45] evaluated the TPs of five different 3D-printed restorative materials for crowns and bridges with a thickness of 1 and 2 mm and exposed to different storage conditions, demonstrating minor changes.

Numerous factors affect the thickness of the restorative material, such as the type of tooth that needs to be repaired, the restorative technique selected, the amount of coverage, and the material’s mechanical and physical characteristics [46]. Dental crown and bridge restorations typically have a 1 to 2 mm thickness, and the material used for temporary prostheses over implant abutments would be slightly thicker [45]. Therefore, the specimens in the current investigation were made with a thickness of 2.5 mm for standardization. The demonstrated TP values in this study showed that the tested materials could produce a more unnatural or opaque outcome compared to enamel. It is well-known that the restoration’s thickness significantly influences the final prosthesis’s color and translucency. An increasing specimen thickness will result in decreased TP values. According to Lambert’s law, less absorption occurs when a material’s thickness is reduced, increasing the amount of light transmission. The thickness of the specimen and the scattering and absorption properties determine the percentage of incident light that is reflected, absorbed, and transmitted [47]. Nevertheless, the outcome of this study should be considered with caution, as the outcome may not necessarily extrapolate to thinner specimens.

Regarding the color changes, the materials in the acid group showed slightly better color stability compared to the water group, except for the T2 material. It has been reported that water absorption is a significant factor in color change susceptibility. Water absorption causes the organic matrix to expand and plasticize, decreasing the material’s composite resin’s durability and promoting color change [30]. Contrarily, the surface of the ceramic particles may be modified by citric acid, which could change their refractive index and lessen the light reflection at the interfaces between the ceramic and the surrounding matrix [48].

Furthermore, two of the three 3D-printed resins, although insignificant, demonstrated slightly better color stability than milled LU material. Visual thresholds are a crucial quality-control instrument for many applications and industries, and dentistry is no exception. Dental and restorative discoloration are clinically significant when they satisfy human visual PT and AT, which are thresholds defining color, TP, and match/mismatch of whiteness in dentistry. Clinical and research results cannot be properly evaluated in terms of real-life relevance without basic comparison with PT and AT, regardless of the results of descriptive and analytical statistics [49]. The PT or “just noticeable difference” refers to the smallest level of color variation that the average observer perceives (CIE Lab 50:50% PT, ΔE  =  1.2). On the contrary, AT refers to the degree of color change that the average observer considers unacceptable, thereby requiring correction deemed acceptable (CIE Lab 50:50% AT, ΔE  =  2.7) [49,50]. In the current study, all the materials, except T2 and LU in water, showed ΔE values below the PT. This infers that the colors of these specimens were visually perceptible compared to the target color (exceeding the PT; ∆E > 1.2). However, the materials demonstrated color changes below the acceptability threshold (ΔE < 2.7). While color matching at or below PT would be ideal, it is often not necessary and can be expensive and time-consuming to achieve a non-perceivable match. However, by complementing its function and meeting patient expectations of an aesthetically pleasing result, the AT value can predict product acceptability in the case of dental restorations [49].

Taşın et al. [31] compared the color changes of three milled (IPS e.max CAD, Vita Enamic, and Cerasmart) and two 3D-printed hybrid ceramic resins (VarseoSmile Crown plus and Permanent Crown) after coffee thermocycling. The outcome showed that the 3D-printed materials had lower color stability compared to milled materials. However, the color change was within the AT, which was in partial agreement with the outcome of the current study. Kim et al. [45] evaluated the ΔE of five different 3D-printed restorative materials for definitive restorations exposed to different storage conditions for 6 months, which demonstrated the differences that varied with the materials tested. The color change at the end of the test period exceeded the AT, which was in disagreement with the outcome of the current study. Similarly, Kurklu et al. [32], comparing the milled and 3D-printed (Permanent Crown Resin) hybrid ceramics, demonstrated that milled resins showed significantly better color stability at one week and one month of storage in different media. Furthermore, Nagai et al. [2] demonstrated a significant difference between the milled and 3D-printed nano-ceramic resins. The authors also showed that the color changes of 3D-printed materials were above the PT and AT.

The low color stability of 3D-printed materials in the previous studies has been related to increased water sorption and hydrolysis. It has been stated that the polymerization rate of 3D-printable materials is rather slow, even after post-curing [51]. Surface softening is associated with an increase in residual monomers due to increased hydrolysis, water sorption, and deteriorating surface integrity [52]. It has been reported that 3D-printed resin-based composites (RBCs) actually displayed a greater water sorption rate than pressed and milled RBCs [53]. Furthermore, fillers are added to enhance the mechanical characteristics of RBCs; they also have an impact on the material’s refractive indices. According to Haffernan and colleagues [54], the masking ability of ceramic-based materials tends to improve with greater thickness, while the TP decreases. Based on the findings of the link between the increase in porcelain translucency and the reduced particle size in its composition, the authors related the differences in the amount of reflected, absorbed, and transmitted light to several factors, including the size of the particles in relation to the wavelength of incident light (0.4–0.7 μm).

When determining the material-specific properties of 3D-printed nanoresin ceramics, the distribution, amount, size, and shape of the fillers are important considerations [19]. The 3D-printed resin’s monomeric content and the size and form of the fillers have been found to influence optical properties [55]. Compared to CAD-CAM-milled nanoresin ceramics, 3D-printed resins require a minimum filler load of 50 wt% to attain satisfactory clinical results [56,57]. In the current study, the 3D-printed products are newly introduced to the market, and the manufacturers maintain control over fabrication. Only a range of filler content or imprecise information about the materials used is frequently provided by manufacturers, who do not always give precise information about their products. Thus, it was not possible to obtain any further information regarding the fillers or the exact composition of the materials tested. Though this study does not fully provide the exact reason for the differences in TP and color based on the inherent composition of the materials, it still provides basic knowledge to clinicians regarding the optical properties of the tested materials.

The study has a few limitations. One of the significant limitations is the build orientation. The materials in the current study were printed at 0°, and other printing angulation was not considered. Different printing angulations could have provided a more thorough understanding of the optical properties of the current study materials. Another significant limitation is the difficulty in obtaining comprehensive information about the constituents used in 3D-printed resins because of intellectual property rights. This limitation is an important hindrance to the thorough assessment and comprehension of the materials used in the current research. The study protocol (immersion, simulated brushing, and ambient temperature) may not completely simulate the in vivo environment. Intra-oral factors such as salivary flow and action, pH, mechanical and chemical hygiene methods could all limit the discoloration process, which was not taken into consideration. The effect of other factors, such as diet and smoking, influencing the optical properties was not considered.

Furthermore, the investigation was carried out in a medium of distilled water and citric acid, which is devoid of chromogenic bacteria and enzymes from the oral environment that can contribute to color change, translucency, and lightness loss. The characteristics of the filler play an important role in light transmittance and thereby affect optical properties. There was no information available regarding the filler content of the 3D printed resins at the reporting time of the study, which is another limitation of the study. However, these factors were not considered during this study. The current study’s scope is confined to three distinct 3D-printed materials from the same manufacturer and one milled control specimen, which may limit how broadly the results can be applied. Finally, the study is limited by a controlled laboratory environment, including the standard thickness, single pH, and specimen shade, which might not be practical in clinical scenarios.

## 5. Conclusions

The translucency of the tested materials was affected by the material type and storage media, while there was no discernible impact on color change, regardless of the material type or the storage media. The translucency of the tested materials varied between 1.96 to 7.51, and the color stability of the tested materials was below the acceptability threshold (2.7 ΔE units). The tested 3D-printed nano-ceramic resins were comparable with milled resins in terms of color stability only.

Future studies should be directed towards evaluating the mechanical and optical properties in clinical scenarios. Furthermore, the current 3D-printed materials should be compared with materials from different manufacturers but with similar applications. This would guide the clinicians in selecting the best material, considering the properties. It would also be interesting to assess and compare the translucency and color changes of these materials with regard to different thicknesses. The optical characteristics of the studied restorative materials might have been better understood by exposure to varying pH levels and should be considered in future studies.

## Figures and Tables

**Table 1:** Details of the study materials.

Resin/Material	Shade/Abbreviation	Manufacturer	Composition	Batch Number
Ceramic Crown	A1/CC	SprintRay Inc., Los Angeles, CA, USA	Mixture of methacrylic acid esters, photoinitiators *, pigment * Inorganic composition includes irregular and some rounded silica (SiO_2_) and ytterbium oxide (Yb_2_O_3_) (1.0–5.0 nm) up to 50% [19]	SRI 0202057
OnX	A1/ONX	SprintRay Inc., Los Angeles, CA, USA	Methacrylate monomers and oligomers, acrylic monomers, inorganic ceramic fillers, photoinitiators *	SRI 0202057
Tough 2	A1/T2	SprintRay Inc., Los Angeles, CA, USA	Mixture of methacrylic acid esters, photoinitiators *, pigment *	SRI 0202164
Lava Ultimate	A1/(LT)/LU	3M ESPE, St. Paul, MN, USA	Nano ceramic fillers (zirconia filler (4–11 nm), silica filler (20 nm), and aggregated zirconia/silica cluster filler) 80 wt%; cross-linked polymer matrix (TEGDMA) 20 wt%	5074A1-LT

* The exact composition is not revealed by the manufacturers.

**Table 2:** Two-way ANOVA to observe the effect of the material and storage media on TP.

Source	Type III SS	df	Mean Square	F	*p*-Value
Corrected Model	215.16	7	30.74	210.99	<0.001 *
Intercept	723.08	1	723.08	4963.49	<0.001 *
Storage media	0.38	1	0.38	2.57	0.12
Materials	213.42	3	71.14	488.33	<0.001 *
Storage media × Materials	1.37	3	0.46	3.13	0.04 *
Error	5.83	40	0.15		
Total	944.07	48			
Corrected Total	220.99	47			

* Statistically significant.

**Table 3:** Mean TP values (Mean ± SD) of the materials at baseline and after simulated brushing.

Storage	Materials	Baseline	Simulated Brushing
Water	Ceramic Crown	2.79 ± 0.28	1.96 ± 0.12
	OnX	2.55 ± 0.28	2.18 ± 0.18
	Tough 2	4.93 ± 0.47	4.21 ± 0.41
	Lava Ultimate	7.49 ± 0.74	7.51 ± 0.64
Acid	Ceramic Crown	3.20 ± 1.11	2.34 ± 0.12
	OnX	2.64 ± 0.38	1.97 ± 0.22
	Tough 2	4.91 ± 0.60	3.80 ± 0.35
	Lava Ultimate	7.23 ± 0.87	7.04 ± 0.59

**Table 4:** Comparison of TP between the materials at different measurement periods.

Time	Storage	Ceramic Crown	OnX	Tough 2	Lava Ultimate	*p*
Baseline	Water	2.79 ± 0.28	2.55 ± 0.28	4.93 ± 0.4	7.49 ± 0.74	<0.001 *
	Acid	3.20 ± 1.11	2.64 ± 0.38	4.91 ± 0.60	7.23 ± 0.87	<0.001 *
Simulated brushing	Water	1.96 ± 0.12	2.18 ± 0.18	4.21 ± 0.41	7.51 ± 0.64	<0.001 *
	Acid	2.34 ± 0.12	1.97 ± 0.22	3.80 ± 0.35	7.04 ± 0.59	<0.001 *
*p* ^†^		*p* > 0.05	*p* > 0.05	*p* > 0.05	*p* > 0.05	

* Statistically significant (*p* < 0.001; one-way ANOVA), ^†^ Independent Student *t* test.

**Table 5:** Two-way ANOVA to observe the effect of the material and storage media on ΔE.

Source	Type III SS	df	Mean Square	F	*p*-Value
Corrected Model	2.27	7	0.33	1.26	0.30
Intercept	57.01	1	57.01	220.92	<0.001 *
Storage media	0.09	1	0.09	0.34	0.56
Materials	1.90	3	0.64	2.46	0.08
Storage media × Materials	0.28	3	0.09	0.37	0.78
Error	10.32	40	0.26		
Total	69.60	48			
Corrected Total	12.60	47			

* Statistically significant (*p* < 0.001).

**Table 6:** Comparison of ΔE (mean ± SD) between materials and storage media.

Parameter	Storage Media	Materials				*p* *
		Ceramic Crown	OnX	Tough 2	Lava Ultimate	
ΔE	Water	0.97 ± 0.85	1.00 ± 0.24	1.28 ± 0.24	1.26 ± 0.62	0.67
	Acid	0.66 ± 0.42	0.98 ± 0.23	1.40 ± 0.34	1.13 ± 0.68	0.07
*p* ^†^		0.45	0.88	0.53	0.74	

* One-way ANOVA; ^†^ Independent Student *t* test.

## Data Availability

The original contributions presented in this study are included in the article. Further inquiries can be directed to the corresponding author.

## Abbreviations

The following abbreviations are used in this manuscript:

3D	Three dimensional
ΔE	Color change
CAD/CAM	Computer-aided design (CAD) and computer-aided manufacturing (CAM)
CIELAB	Commission Internationale de l’Éclairage Lab color space
CC	Ceramic Crown
LU	Lava Ultimate
OnX	SprintRay OnX
T2	Tough 2
SPSS	Statistical Package for the Social Sciences
TP	Translucency
